# The potential of lactic acid bacteria isolated from *ikan budu* (fermented fish) to inhibit the growth of pathogenic fungi and detoxify aflatoxin B1

**DOI:** 10.14202/vetworld.2023.1373-1379

**Published:** 2023-07-04

**Authors:** Yetti Marlida, Nurmiati Nurmiati, Husmaini Husmaini, Nurul Huda, Lili Anggraini, Laily Rinda Ardani

**Affiliations:** 1 Department of Animal Nutrition, Faculty of Animal Science, Andalas University, Limau Manis Campus, Padang 25163, West Sumatra, Indonesia; 2 Department of Biology, Faculty of Mathematics and Natural Sciences, Andalas University, Padang City, West Sumatra 25175, Indonesia; 3 Department of Animal Production, Faculty of Animal Science, Andalas University, Limau Manis Campus, Padang 25163, West Sumatra, Indonesia; 4 Faculty of Sustainable Agriculture, Universiti Malaysia Sabah, 90509, Sandakan, Sabah, Malaysia; 5 Graduate Program of Animal Science, Andalas University, Limau Manis Campus, Padang 25163, West Sumatra, Indonesia

**Keywords:** biodetoxification, feedstuffs, lactic acid bacteria, pathogenic fungi

## Abstract

**Background and Aim:**

Market demand for safe feed and food supply and consumer preferences for safe and healthy products are increasing. Control measures to counter threats to the feed supply need to be implemented as early as possible to prevent economic losses. Mycotoxins produced by certain groups of fungi are a problem that can disrupt the feed supply or pose a threat to the health of animals and humans. Biological control to detoxify contaminated feed ingredients can be carried out on a large scale economically. For example, lactic acid bacteria (LAB) can act as biological agents for eliminating mycotoxins. This study aimed to clarify the value of screening LAB to inhibit *Aspergillus flavus* growth and detoxify aflatoxin B1 (AFB1).

**Materials and Methods:**

In this study, using a completely randomized design with three replications, five isolates of LAB (LA.1, LA.6, LA.8, LA.12, and LA.22) along with their supernatants were tested qualitatively and quantitatively for their ability to counter mycotoxins using *A. flavus* and corn kernels. The isolates with the best activity were identified by sequencing 16S rDNA.

**Results:**

The results showed that the five LAB isolates can inhibit the growth of *A. flavus* and detoxify AFB1. Among these isolates, LA.12 showed the best performance, followed by LA.22, LA.8, LA.6, and then LA.1. The sequencing results confirmed that LA.12 was *Lactobacillus harbinensis* strain 487.

**Conclusion:**

All of the isolates in this study have the potential as biological agents for detoxifying AFB1, with isolate LA.12 appearing to be the most promising biodetoxification agent for feed (AFB1 in corn) based on its ability to inhibit pathogenic fungi.

## Introduction

A major problem in corn farming is contamination by the fungus *Aspergillus flavus*, which can produce aflatoxins. Such toxins decrease corn product quality and prevent its acceptance by large companies. Aflatoxins also impact the quality of livestock products such as meat, eggs, and milk, adversely affecting consumer preferences. *Aspergillus flavus* is naturally found in soil, grains (corn and soybeans), and feed. Grain crops are readily contaminated by *A. flavus* in the growing and post-harvest periods, both in storage and during distribution [1]. Besides impacting human health, animal health, and productivity, mycotoxins also impact domestic and international trade [2, 3]. According to the Food and Agriculture Organization, approximately 25% of food and feed are contaminated by mycotoxins produced by fungi [4].

Mycotoxins are secondary metabolites of fungi that grow on various foods and feed commodities at various stages of the production process. Biological contamination from molds of *Aspergillus*, *Penicillium*, and *Fusarium*, which are mycotoxin producers, is a serious concern because of the threat they pose to the health of livestock and humans. The negative impacts of mycotoxins on health in humans, birds, and primates include mutagenic, teratogenic, and carcinogenic effects [5]. *Aspergillus* flavus, *Aspergillus parasiticus* and a small part of *Aspergillus nomius* are molds that produce aflatoxin B1 (AFB1), which is a natural contaminant of food/feed products and most frequently found in feed. It has potent mutagenic and carcinogenic effects. Aflatoxins can accumulate in the livestock body, passing through the bloodstream and entering the tissues, contaminating animal products such as milk, eggs, and meat. This poses a health hazard to consumers [6].

Studies have attempted to reduce aflatoxins in granulated feed through antifungal compounds produced by the following lactic acid bacteria (LAB): *Lactobacillus plantarum* [7], *Lactobacillus casei*, *Lactobacillus acidophilus*, and *Lactobacillus paracasei* isolates [8]. In another study, bacterial isolation from fermented fish products (*budu*) from two areas in West Sumatra, namely, Sungai Limau, Padang Pariaman Regency, and Air Bangis, Pasaman Regency, was carried out; metagenomic analysis on the samples showed the dominance of LAB [9]. Screening results showed five candidate probiotic isolates that were highly capable of surviving at low pH (pH 2.0) and in the presence of 0.3% bile salts and could kill pathogenic bacteria such as *Escherichia coli*, *Salmonella* Enteritidis, and *Staphylococcus aureus* [10]. These isolates have the potential as inhibitors of pathogenic fungi and detoxification of aflatoxin B1, but there has been no research on this.

Lactic acid bacteria use several mechanisms to reduce the level of AFB1. For example, the detoxification of feed granules containing AFB1 by LAB is accomplished using live microbial cells and/or enzymes produced by certain LAB strains [11]. Bioactive metabolites produced by LAB can inhibit fungal growth and prevent the production of mycotoxins in food and feed. The bioactive compounds produced include acids, carbon dioxide, hydrogen peroxide, phenyl lactic acid, azelaic acid, and bioactive peptides with a low molecular weight [12]. It has also been reported that the proteolytic enzymes produced by LAB play a role in detoxifying mycotoxins in food and feed [13]. Lactic acid bacteria produce proteolytic enzymes that hydrolyze proteins (including cell wall proteins) into polypeptides, peptide transporters that transfer peptides into cells and intracellular peptidases that degrade peptides into amino acids [14]. Moreover, the enzyme aflatoxin-detoxifizyme was isolated and purified from the pathogenic fungus *Armillaria tabescens* and showed detoxification activity against AFB1 [14].

Based on previous studies [9, 10] conducted on the role of LAB in inhibiting the growth of pathogenic fungi and reducing AFB1 in granulated feed, especially corn, a preliminary study was conducted to investigate such abilities of five LAB strains. These strains had previously been proven to act as probiotics for livestock, but had never been tested for their antifungal/AFB1 effects.

## Materials and Methods

### Ethical approval

Ethical approval was not required because this study did not used any live animals.

### Study period and location

This study was conducted from August to December 2022 at the Feed Industry Technology Laboratory, Faculty of Animal Sciences, Andalas University, Padang, West Sumatera, Indonesia.

### Lactic acid bacteria

The LAB used in this study were five LAB isolates from fermented fish (*budu*), which were selected as probiotic candidates and recultured in an industrial feed technology laboratory [10].

### Preparation of fungal spore solution

Fungal spores were prepared in accordance with a slightly modified version of the method of Yang and Chang [15]. The test fungus used was *A. flavus*, which had been isolated from moldy corn and identified at the Biology Laboratory of the Faculty of Natural Sciences and Mathematics, Andalas University, Padang, West Sumatera, Indonesia. The mold was grown in potato dextrose agar medium (Merck, Darmstadt, Germany) at 30°C for 7 days until sporulation occurred. Spores were harvested from the medium by adding sterile distilled water containing 0.05% (v/v) Tween 80 and shaking gently. The fungal spores used were stored at −20°C in glycerol: water (20:80). Spore concentration in the mold was determined using a hemocytometer with a concentration of 10^7^ spores/mL of solvent.

### Qualitative analysis of antifungal activity

Qualitative analysis of the antifungal activity was carried out in accordance with Strom’s method [16]. The medium used for the growth of *A. flavus* was PDA with a mold spore concentration of 10^6^ spores/mL. Lactic acid bacteria isolates were chosen to detect the presence of antifungal activity with a streak plate method on de man rogosa sharpe agar (Merck). *Aspergillus flavus* was inoculated on the medium using the spread plate method, and the LAB isolates were subsequently inoculated by the streak plate method. The results of inoculation were grown under anaerobic conditions for 24 h at a temperature of 37°C. After incubation, if a clear zone was formed in the medium, the LAB isolates were considered to have antifungal activity and were reselected for the next screening.

### Quantitative analysis of antifungal activity

Quantitative analysis of antifungal activity was carried out using the diffusion method in accordance with the method of Yang and Chang [15]. The first step involved the preparation of cell-free supernatant from LAB. Selected LAB were grown in MRS Broth medium at 37°C for 24 h. Cultures were centrifuged at 9500× *g* for 15 min and subsequently passed through a filter with a pore size of 0.45 μm. The obtained cell-free supernatant was then used for testing. Each test was carried out in triplicate. The medium used for testing was PDA in a dish supplemented with 10^6^ mold spores/mL medium (20 mL of PDA) containing 1.5% (w/v) agar. In the paper test, disks with a diameter of 8 mm were placed in a PDA cup, and then added 100 μL of the cell-free supernatant sample dropwise The plates were incubated at 30°C for 48 h and the diameter of the inhibition zone formed around wells or disc paper was measured using Vernier calipers.

### Screening of LAB capable of detoxifying AFB1

Five LAB isolates (LA.1, LA.6, LA.8, LA.12, and LA.22) were investigated to determine their ability to reduce AFB1. Coumarin medium (CM) was used to evaluate the ability to reduce AFB1 on culture at 37°C for 48 h [17]. Coumarin (1%) was used as the only carbon source in CM. Isolates that could grow in CM were considered able to degrade AFB1.

### Optimization of AFB1 removal by LAB

The degradation of AFB1 by the five different isolates was tested on corn kernels. The isolate culture was incubated at 30°C and 12× *g* for 48 h in an incubator shaker. After incubation, the cells were sprayed on corn kernels and incubated at 27°C for 24 h. Corn kernels containing aflatoxin emitted a distinctive greenish fluorescent color when exposed to ultraviolet light at a wavelength of 365 nm.

### Identification of selected promising LAB

Selected promising LAB strains were identified by 16S rDNA sequence analysis. The template DNA of these strains was extracted [18], and 16S rDNA was amplified using universal primers 27F (5’-AGAGTTTGATCCTGGCTCAG-3’) and 1492R (5’-TACGGCTACCTTGTTACGACTT-3’). Polymerase chain reaction amplification products were separated using 1.0% agarose gel electrophoresis at 80 V for 30 min. 16S rDNA sequence analysis was performed at Sangon Biotechnology Co., Ltd. (Shanghai, China). The sequence results were analyzed using the GenBank NCBI BLAST database (Basic Local Alignment, http://blast.ncbi.nlm.nih.gov).

### Statistical analysis

This study used a completely randomized design with experiments performed in triplicate for each stage of the study. Data obtained from this study were subjected to analysis of variance using Statistical Package for the Social Sciences (SPSS) software version 21.0 (IBM SPSS Statistics, USA). If a significant difference in the mean was identified (p < 0.05), *post hoc* Tukey’s HSD test was subsequently performed.

## Results

### Antifungal activity of LAB

**Figure-1:**
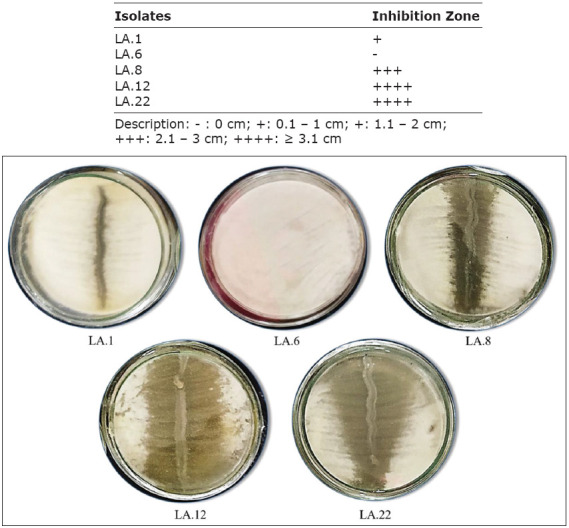
Qualitative analysis: antifungal activity of several lactic acid bacteria isolates against *Aspergillus flavus* using the overlay method.

Based on the initial qualitative screening using the overlay method, as shown in Figure-1, some LAB isolates were found to have antifungal ability, especially isolates LA.12, LA.22, and LA.8. Meanwhile, other isolates, such as LA.1, only showed low antifungal activity and isolate LA.6 showed none at all.

**Figure-2:**
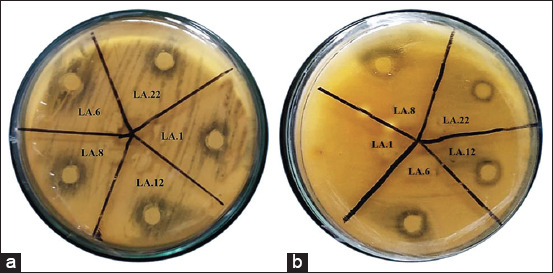
Quantitative analysis: antifungal activity of several lactic acid bacteria isolates against *Aspergillus flavus* with the streak plate method (a) supernatant, (b) biomass.

**Figure-3:**
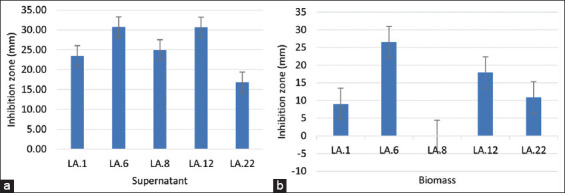
Antifungal activity of several lactic acid bacteria isolates against *Aspergillus flavus* with the paper disc diffusion method, (a) supernatant, (b) biomass.

Further testing of promising LAB isolates by the paper disc diffusion method (quantitatively) using the resulting supernatant (Figure-2a) and cell-free supernatant (Figure-2b) was performed because each strain has a different ability to inhibit fungal growth (Figure-3). Isolate LA.6 showed the greatest inhibition of fungal growth by the supernatant (30.70 mm); this value did not differ significantly (p > 0.05) from that of isolate A12 (30.62 mm) and was highly significantly different (p < 0.01) from the inhibition zones of isolates LA.8, LA.1, and LA.22 (Figure-3a).

The results of testing the biomass of LAB isolates as antifungals differed slightly with the use of supernatants. Isolate LA.6 showed the greatest inhibition of fungal growth by biomass (26.5 mm), which was highly significantly different (p < 0.01) from the inhibition zones of isolates LA.12, LA.22, and LA.1 (Figure-3b). In this test, the biomass for isolate LA.8 did not show signs of inhibition of the growth of *A. flavus*.

### Biodetoxification of AFB1 by LAB

**Figure-4:**
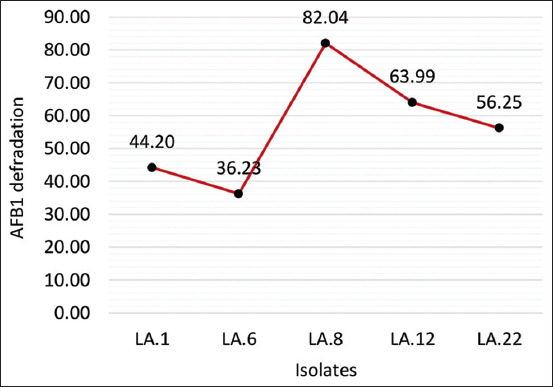
Detected the ability of several lactic acid bacteria isolates to degrade aflatoxin B1 using coumarin medium.

The results revealed highly significant differences among the five strains of LAB tested regarding the removal of AFB1, as shown in Figure-4 (p < 0.01). After further testing, it was found that the LA.8 strain could eliminate AFB1 at the highest rate of 82.04%, followed by LA.12, LA.22, LA.1, and then LA.6.

### Biodegradation of AFB1 by LAB isolates

**Figure-5:**
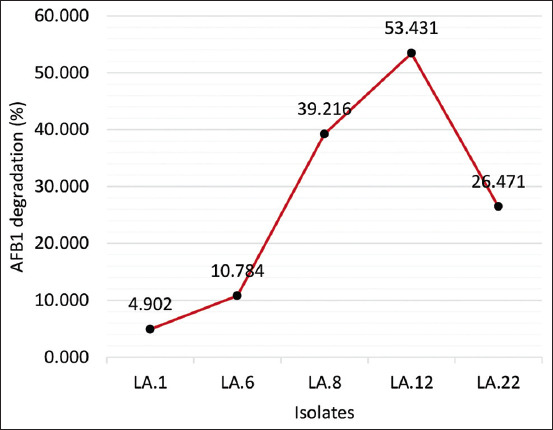
Detected the ability of several lactic acid bacteria isolates to degrade aflatoxin B1 using corn kernels.

The results of testing the ability of the five LAB isolates to degrade AFB1 showed a highly significant difference among them (p < 0.01) (Figure-5). Isolate LA.12 was best at degrading AFB1 at a rate of up to 53.43%, followed by isolates LA.8, LA.22, LA.6, and then LA.1.

### Identification of LA.12 isolate as a promising LAB

**Figure-6:**
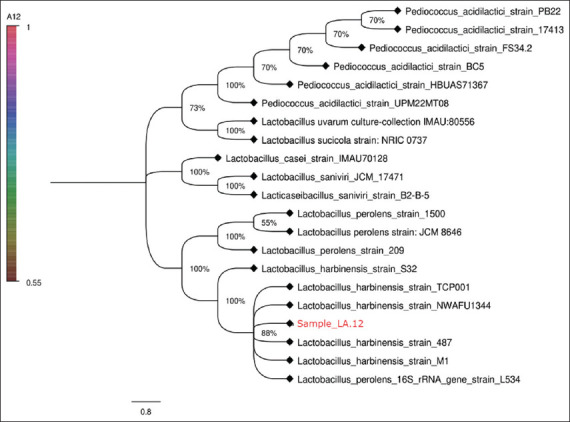
Phylogenetic tree of LA.12 using the neighbor-joining method.

The promising strain LA.12 was identified by 16S rDNA sequence analysis. The analysis results from LA.12 in Figure-6, sequences in the GenBank NCBI BLAST database show the phylogenetic tree using the neighbor-joining method.

## Discussion

### Antifungal activity of LAB

Figures-1 and 2 show the partial inhibition zone is an area of tested fungi that experiences thinning, while the total inhibition zone is a clear area around the isolate that is created from antifungal activity by bioactive compounds capable of killing fungi. Each LAB strain analyzed in this study differed in its ability to inhibit the growth of *A. flavus*. Some indigenous microorganisms found in food can prevent the adverse effects of mycotoxins on health [19, 20]. Lactic acid bacteria strains have antifungal properties due to several factors, such as competition with fungi for growth, lowering pH, and the production of compounds with antifungal properties such as organic acids, protein compounds, fatty acids, and substances such as bacteriocins [21]. In line with the results of this study, previous studies showed that *Lactobacillus* can inhibit the production of AFB1 and the growth of *Aspergillus* spp. [22–24].

Organic acids are considered to affect the growth of herbal medicine by inhibiting the growth of mycelium [25]. Lactic acid bacteria produce organic acids that can lower the pH of the medium. Lactic acid bacteria have an advantage in the speed of reacting to changes in the pH of the medium to minimize acid stress and prevent it from interfering with their metabolic processes. On the other hand, *A. flavus* must expend more energy coping with changes in environmental pH and adapting its metabolic processes [26]. This makes *A. flavus* undergo growth inhibition.

Lactic acid bacteria inhibit the growth of fungi by producing metabolites, including organic acids, cyclic peptides, reuterin, and hydrogen peroxide [25]. The most widely reported antifungal activity of LAB involves organic acids produced from the primary carbohydrate fermentative metabolism. Other studies reported the production of organic acids having antifungal properties, such as lactic acid, acetic acid, formic acid, propionic acid, butyric acid, phenylacetate, and hydroxyphenylacetate [15, 21, 27, 28]. Lactic acid bacteria also produce proteolytic enzymes that can hydrolyze proteins, including cell wall proteins into polypeptides, peptide transporters that transfer peptides into cells, and intracellular peptidases that can degrade peptides into amino acids [14].

*Lactobacillus* spp. produces antifungal compounds, namely, methylhydantoin and cyclic dipeptides cyclo; these active compounds were reported to play roles in inhibiting aflatoxin production by *A. flavus* and *A. parasiticus* [29]. The pH of the medium decreases due to the production of organic acids, so the environmental conditions are not conducive to the growth of pathogenic microorganisms [30]. For example, Guimaraes *et al*. [21] reported 50% inhibition of the growth of the fungus *Penicillium nordicum* with LAB supernatants containing organic acids.

### Biodetoxification of AFB1 by LAB

All aflatoxins have a basic molecular structure in the form of a coumarin [27, 31]. Microorganisms with the ability to use coumarins as a carbon source are also thought to be able to use aflatoxins, in this case, AFB1 [32]. Aflatoxins include bisfuranokumarin derivatives and a lactone ring in the coumarin structure, which play important roles in their toxicity and mutagenicity [33]. Guan *et al*. [32] found 65 LAB isolates that could reduce AFB1 concentrations with media containing coumarin, including the following: *Stenotrophomonas maltophilia*, *Bacillus* spp., *Brevundimonas* spp., *Bacillus* spp., *Klebsiella* spp., *Brevundimonas* spp., *Enterobacter* spp., *Brachybacterium* spp., *Rhodococcus* spp., and *Cellulosimicrobium* spp.

The main processes in the degradation of AFB1 consist of cleavage of lactone groups or modification of difuran rings or coumarin structures [32]. Several enzymes involved in AFB1 degradation are laccase, peroxidase, oxidase, and reductase [34]. Lactic acid bacteria produce organic acids such as hydrogen peroxidase, which have been proven effective for biocontrol [35], and LAB such as *Pediococcus acidilactici* produce laccase [36].

### Biodegradation of AFB1 by LAB isolates

The ability of LAB to act as biodegradation agent is influenced by the LAB type. The LAB type can reflect the particular biochemical activity of the bacteria. The main products of LAB are lactic acid, organic acids, hydrogen peroxide, and bacteriocins, which act as antimicrobials [37]. Lactic acid is produced as a primary metabolite and is a growth-associated product [38]. Lactic acid bacteria promote the acidification of raw materials during fermentation and produce organic acids, CO_2_, H_2_O_2_, fatty acids, antifungal peptides, volatile compounds, and other antifungal compounds [39].

The biological process of mycotoxin detoxification by LAB consists of chemical/enzymatic degradation, metabolic conversion, and adsorption. Mycotoxins are bound by LAB cell wall components, preventing them from contaminating food or feed [40].

### Identification of LA.8 isolate as potential LAB

The phylogenetic tree shows the LA.12 kinship relationship (Figure-6). The phylogenetic tree is known to show a high consistency of the relationships between organisms. The bootstrap value is used to determine the reliability of phylogenetic tree reconstruction. The phylogenetic tree presented in Figure-6 shows the LA.12 kinship relationship, indicating high consistency of the relationship between organisms.

**Figure-7:**
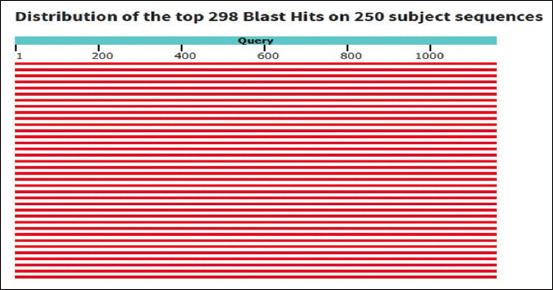
Graphic summary of LA.12 and *Lactobacillus harbinensis* strain 487.

Isolate LA.12 has similarity of 100% in 100% query coverage with *Lactobacillus harbinensis* strain 487. This value indicates that the isolate can be considered as the same species with *L. harbinensi*s strain 487. The sequence has a high degree of homology, as indicated by the red results with a score of ≥200 (Figure-7). Based on this homology, it can be concluded that the two sequences are the same and have an evolutionary relationship.

## Conclusion

All of the isolates analyzed in this study may be used to biodetoxify AFB1, with isolate LA.12 being the most promising novel agent for the biodetoxification of feed (AFB1 in corn). It could be particularly valuable given its ability to inhibit pathogenic fungi. LA.12 was identified here as *L. harbinensis* strain 487.

## Authors’ Contributions

YM and NN: Supervised the study and drafted the manuscript. HH and NH: Analyzed data and revised the manuscript. LA and LRA: Laboratory work. All authors have read, reviewed, and approved the final manuscript.
